# Small phytoplankton contribute greatly to CO_2_-fixation after the diatom bloom in the Southern Ocean

**DOI:** 10.1038/s41396-021-00915-z

**Published:** 2021-03-12

**Authors:** Solène Irion, Urania Christaki, Hugo Berthelot, Stéphane L’Helguen, Ludwig Jardillier

**Affiliations:** 1 Université Littoral Côte d’Opale - ULCO, CNRS, Université Lille, UMR 8187 - LOG - Laboratoire d’Océanologie et de Géosciences, F-62930 Wimereux, France; 2 Laboratoire des Sciences de l’Environnement Marin (LEMAR), UMR 6539 UBO/CNRS/IRD/IFREMER, Institut Universitaire Européen de la Mer (IUEM), Brest, France; 3 Ecologie Systématique Evolution, Université Paris-Saclay, Centre National de la Recherche Scientifique - CNRS, AgroParisTech, Orsay, France

**Keywords:** Biogeochemistry, Biogeochemistry, Stable isotope analysis, Microbial ecology

## Abstract

Phytoplankton is composed of a broad-sized spectrum of phylogenetically diverse microorganisms. Assessing CO_2_-fixation intra- and inter-group variability is crucial in understanding how the carbon pump functions, as each group of phytoplankton may be characterized by diverse efficiencies in carbon fixation and export to the deep ocean. We measured the CO_2_-fixation of different groups of phytoplankton at the single-cell level around the naturally iron-fertilized Kerguelen plateau (Southern Ocean), known for intense diatoms blooms suspected to enhance CO_2_ sequestration. After the bloom, small cells (<20 µm) composed of phylogenetically distant taxa (prymnesiophytes, prasinophytes, and small diatoms) were growing faster (0.37 ± 0.13 and 0.22 ± 0.09 division d^−1^ on- and off-plateau, respectively) than larger diatoms (0.11 ± 0.14 and 0.09 ± 0.11 division d^−1^ on- and off-plateau, respectively), which showed heterogeneous growth and a large proportion of inactive cells (19 ± 13%). As a result, small phytoplankton contributed to a large proportion of the CO_2_ fixation (41–70%). The analysis of pigment vertical distribution indicated that grazing may be an important pathway of small phytoplankton export. Overall, this study highlights the need to further explore the role of small cells in CO_2_-fixation and export in the Southern Ocean.

## Introduction

Carbon fixation (CO_2_-fixation) by marine phytoplankton accounts for about half the Earth’s primary production [1–3]. Some 20% of phytoplankton’s net primary production (5–10 Gt C) is exported to the deep ocean via the biological pump [4, 5]. The magnitude and nature of the carbon exported to the deep ocean is impacted by the size-structure of phytoplankton communities [6, 7]. High carbon export (C-export) out of the photic zone is classically linked to the dominance of large phytoplankton (herein defined as >20 µm cells) because of their high sinking velocity or packaging into dense fecal pellets produced by large grazers [8–10]. Alternatively, it has been suggested that small phytoplankton contribution to export was proportional to their total net primary production through aggregation into larger sinking particles, or, as for their larger counterpart, export as fecal pellets produced by higher trophic levels [11]. Determining the contribution of diverse phytoplankton size-groups to CO_2_-fixation is thus the first step to characterize the functioning of the carbon pump. This is routinely achieved by measuring size-fractionated CO_2_-fixation rates in natural communities using isotopic tracers (^14^C or ^13^C labeled substrates) that can be used to model marine production [12, 13]. Defining a general size-scaling relationship for CO_2_-fixation from the smallest autotrophic cells to large microbial eukaryotes based on accurate measurements represents thus a major challenge for models relying on such theoretical relationships for phytoplankton growth modeling [14, 15]. Moreover, size-based models may be biased due to metabolic variability within a size-class grouping diverse phylogenetic taxa [16, 17]. Including phylogenetic features can further refine phytoplankton production models, but increase drastically their complexity [18–20]. Consequently, it is crucial to determine the degree of complexity required (e.g., size, species, population) and define the descriptors (e.g., biomass, abundances) needed to improve models. To do so, in situ measurements are required to appreciate the variability of CO_2_-fixation rates between and within different phytoplankton groups. Diverse studies have previously revealed that the contribution to biogeochemical cycles is not necessarily proportional to microbial group abundance or biomass. For example, flow cytometry sorting of different small-sized microbial autotrophs has revealed that although picoeukaryotes are far less abundant than cyanobacteria, their contribution to CO_2_-fixation is similar or even greater [21–23]. More recently, secondary ion mass spectrometry (SIMS) has allowed measurements at the single-cell scale at a resolution of ~50 nm (NanosSIMS) or 1 μm (large geometry SIMS). A pioneer lacustrine study revealed that rare phototrophic bacterial taxa (0.3% of the total cell number) could contribute to more than 70% of the total carbon uptake [24]. Subsequent studies also revealed higher marine phytoplankton contribution to C- or N-fixation than expected from their relative abundance or biomass for diverse microbial groups. This has been observed for example for diazotroph-associated diatoms [25], chain-forming diatoms [26], or specific pico-phytoplankton groups [27]. This approach also unveiled a high microbial intra-group heterogeneity in C- or N-uptake, likely affecting the group’s adaptation potential to changing environments [24, 25, 27–29]. The reasons for this high heterogeneity are unclear but could result from intra-group genetic diversity, intra-group differences in gene expression, or cell life history [24]. Recently, it has also been suggested that intra-specific variability in C- or N-uptake is correlated to differences in biovolumes from one cell to the other [30].

The Southern Ocean (SO) contributes up to 40% (42 ± 5 Pg C over the period 1861–2005) of the oceanic uptake of anthropogenic CO_2_ [31, 32]. It is an ideal study area to explore phytoplankton CO_2_-fixation by contrasted phytoplankton communities. Most of the SO is composed of high-nutrient, low-chlorophyll (HNLC) areas, where primary production is limited by iron despite high macronutrients concentrations [33–35]. In these low-productive environments, phytoplankton communities and primary production are typically dominated by small cells (<20 µm) [36, 37]. However, large diatoms have attracted most attention because of the enhanced production and C-export observed during diatom blooms in discrete, naturally iron-fertilized regions of the SO, such as Kerguelen, Crozet, or South Georgia during spring and summer [38–40]. This study is part of the MOBYDICK cruise (Marine Ecosystem Biodiversity and Dynamics of Carbon around Kerguelen: an integrated view) that aimed at understanding the link between biodiversity and carbon fluxes on and off the naturally iron-fertilized Kerguelen plateau. Off-plateau, phytoplankton biomass and production are dominated throughout the year by small size-groups [12, 41]. On-plateau, spring blooms of chain-forming and large diatoms typically end in February because of silicic acid and iron co-limitation [42]. MOBYDICK was the first study in this area that took place after the diatom bloom (March 2018).

Our main objective was to describe the diversity and assess the role of small phytoplankton in CO_2_-fixation in post-bloom conditions. Surface CO_2_-fixation and division rates of phytoplankton at the single-cell level were measured, focusing on small phytoplankton (non-silicified and small diatoms), which have been overlooked so far in this Oceanic region. Changes in the contribution of broad taxonomic groups to chlorophyll *a* (Chl *a*) with depth were used to discuss how small phytoplankton could potentially contribute to C-export.

## Materials and methods

### Sampling location

**Fig. 1:**
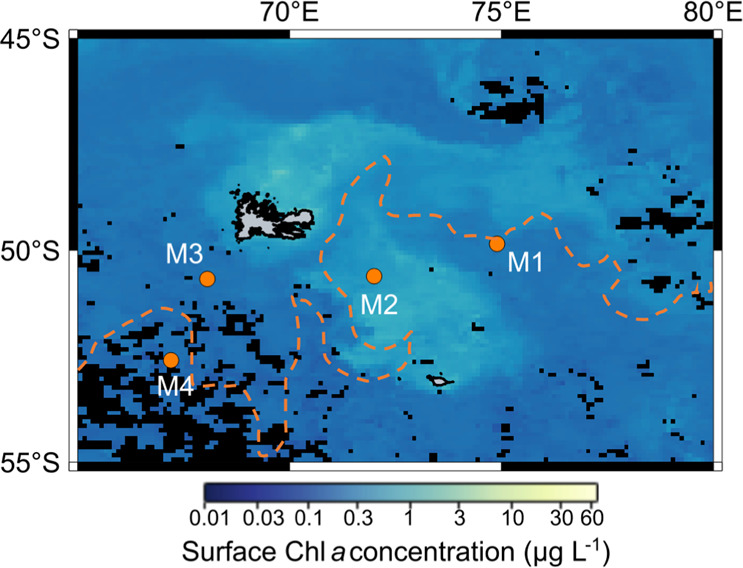
Map of the study area. Surface chlorophyll *a* concentrations correspond to AQUA/ MODIS average values for March 2018. The orange dashed line indicates the position of the polar front after Pauthenet et al. [88].

Four different sites were visited on and off the Kerguelen Plateau during the MOBYDICK cruise (Table 1). Station M2, located on the iron-fertilized plateau, was sampled three times at 9–10-day intervals. This station corresponds to the “historical” plateau reference station of KEOPS1 and KEOPS2 cruises A3. This station was considered as characteristic of iron-fertilized plateau waters, with long residency time and an eddy-like structure [43]. Three off-plateau stations were also sampled (M1, M3, and M4). Station M4 was sampled twice at 2-week intervals, and M1 and M3 were sampled only once. M2, M1, and M4 were located south of the polar front in Antarctic waters. M3 was located south-west of the plateau in subantarctic waters (Fig. 1). Samples were collected with a rosette equipped with Niskin bottles and a CTD probe (SeaBird 911-plus). Three casts were done at all stations for: (1) nutrient concentration measurements as well as phytoplankton community composition based on pigment analyses; (2) microbial eukaryote community composition through a metabarcoding approach: and (3) CO_2_-fixation measurements using stable isotope (^13^C) tracer experiments.

**Table 1:** Main surface biogeochemical parameters of the stations sampled.

	Station	Sampling date	Temperature (°C)	NH_4_^+^ (nmol L^−1^)	NO_3_^−^ (µmol L^−1^)	PO_4_^3−^ (µmol L^−1^)	Si(OH)_4_ (µmol L^−1^)	Mixed layer depth (m)	Euphotic layer (m)	Chl *a* (µg L^−1^)
Off-plateau	M1	09/03/2018	5.08	421	24.8	1.63	6.49	27	80	0.31
	M3_1	05/03/2018	5.60	501	23.4	1.62	2.31	65	93	0.19
	M4_1	01/03/2018	4.49	354	25.5	1.72	4.13	49	96	0.18
	M4_2	12/03/2018	4.47	481	24.8	1.71	4.80	87	100	0.22
Plateau	M2_1	26/02/2018	5.21	704	21.6	1.45	1.17	62	64	0.28
	M2_2	07/03/2018	5.24	1090	21.3	1.47	1.29	61	61	0.32
	M2_3	17/03/2018	5.18	899	21.8	1.51	2.60	68	58	0.58

Nutrients and Chl *a* concentration indicated in the table were sampled at 10 m for plateau stations and 25 m for off-plateau stations. The mean mixed layer depth (difference in sigma of 0.02 to the surface value) and euphotic layer depth (1% light depth) of all CTD casts performed during the occupation of the stations is given.

### Water sampling for nutrients and pigment analysis

Samples for dissolved inorganic nutrients measurements (silicic acid, nitrate, phosphate, and ammonium) and pigment analysis were taken at all stations at 9–10 depths (10, 25, 50, 75, 100, 125, 150, 175, 200, and 250 m). Ammonium was measured by fluorometry [44]. Other nutrients were analyzed colorimetrically as described in Aminot and Kérouel [45].

For pigment analysis, 2.3 L of seawater were collected and filtered onto Whatman GF/F filters. Filters were then flash-frozen in liquid nitrogen and stored at −80 °C. Pigment determination was done using High Performance Liquid Chromatography (HPLC), following the method of Ras et al. [46]. HPLC data from all sampling points in the first 250 m were considered for CHEMTAX analysis [47]. The contribution to Chl *a* of seven different taxonomic groups (chlorophytes, prasinophytes, cyanobacteria (*Synechococcus* spp.), cryptophytes, diatoms, autotrophic dinoflagellates (with peridinin), and haptophytes (*Phaeocystis* like)) was determined based on their characteristic pigment profiles. Samples were first clustered based on their pigment concentration ratios to form homogeneous bins. Then, pigment:Chl *a* ratios were adjusted for each bin using a 60 randomized ratio matrix varying by up to 35% of the initial ratio matrix to avoid any bias linked to the ratios chosen from the literature [48, 49]. The contribution of the different groups to Chl *a* was determined by averaging the six best runs.

Phaeopigments (the sum of phaeophytin-*a* and phaeophorbide-*a*) are degraded Chl *a* products. Phaeophytin-*a* is traditionally thought to result from grazing, while phaeophorbide-*a* may arise from both phytoplankton senescence and grazing [50]. In this study, it was considered that phaeopigments were mostly likely associated with grazing activity [51], since chlorophyllide-*a*, a degradation pigment associated with cell senescence [48], was only detected at very low concentration (<0.004 µg L^−1^) in two samples. The ratio phaeopigments:Chl *a* was determined from the surface down to 250 m. A ratio <1 indicates that phytoplankton material is mostly fresh, whereas a ratio >1 indicates mostly degraded material [52].

### Water sampling for metabarcoding of phytoplankton communities

Water samples were collected at 15 m at all stations to describe small and large phytoplankton communities with metabarcoding of the 18S rRNA gene. After pre-filtering through 100 μm nylon mesh (Milipore, USA) to remove most of the metazoans, 10 L of seawater were successively filtered through 20 μm nylon mesh (Millipore, USA) using 47 mm diameter Swinnex (Millipore, USA) and 0.2 μm Isopore polycarbonate filters (Millipore, USA) using 90 mm diameter inox filtration systems. Filters were stored at −80 °C until processing. DNA was extracted following PowerSoil DNA Isolation Kit (QIAGEN, Germany) standard manufacturer’s protocol. The 18S rRNA gene V4 region was amplified using EK-565F (5′-GCAGTTAAAAAGCTCGTAGT) and UNonMet (5′-TTTAAGTTTCAGCCTTGCG) primers [53]. Pooled samples were paired-end sequenced (2 × 300 bp) on a MiSeq (Illumina, San Diego, CA, USA) at the company Genewiz (South Plainfield, NJ, USA). Quality filtering of the reads, identification of amplicon sequencing variants (ASV) and taxonomic affiliation based on the PR2 database v.4.11 [54] were done in the R-package DADA2 [55]. ASVs affiliated to divisions Chlorophyta, Cryptophyta, Haptophyta, Ochrophyta, and class marine ochrophytes were filtered to describe phytoplankton communities. Relative abundances of ASVs were normalized to the total number of sequences affiliated to autotrophic phylogenetic taxa to build relative abundance heatmaps of small (0.2–20 µm) and large (20–100 µm) phytoplankton taxa with package ampvis2 [56]. Raw sequencing files in fastq format, as well as ASVs, taxonomy, and metadata tables are available on NCBI (accession numbers SAMN17058185- SAMN17058198).

### Water sampling for stable isotope experiments

Seawater samples were collected at each site at least 1 h before sunrise from surface waters (10 or 15 m depth) to evaluate phytoplankton CO_2_-fixation. Five HCl-cleaned polycarbonate 12.5 L carboys were filled with 12 L of seawater prefiltered on a 100 µm mesh (Fig. S1). Three carboys were spiked with 12 mL of NaH^13^CO_3_ solution (99% ^13^C, Cambridge Isotope Laboratories, Inc.), targeting an enrichment of 10% in DI^13^C. Two carboys were left unspiked as negative control at T_0_ and T_final_. Four carboys (one control and three carboys enriched with DI^13^C) were incubated on-deck from dawn to dusk (Table S1). In situ temperature was reproduced in the incubator by a constant flow of sub-surface seawater. In situ light intensity of the sampling depth was mimicked using blue light screens attenuating direct sunlight by ~50%. Among the three ^13^C enriched carboys, one was left in the dark. Incubations were stopped after sunset by adding paraformaldehyde (PFA; 1% final concentration w/v). After 1 h of fixation in the dark, several sub-samples were taken:

1. To calculate the bulk CO_2_-fixation of the community, triplicates from each carboy of 1.5 L were filtered onto precombusted (450 °C, 4 h) GF/F filters, rinsed three times with 20 mL of filtered seawater (0.2 µm pore size membranes) and stored in precombusted dark glass tubes at −80 °C. Back in the laboratory, these filters were dried at 60 °C overnight, pelletized into tin capsules and analyzed by an elemental analyzer coupled to a continuous flow isotope-ratio mass spectrometer (EA-IRMS).
2. To measure the CO_2_-fixation at the single-cell level (large geometry SIMS and nanoSIMS analysis), large (>20 µm) and small (<20 µm) cells were collected in duplicates for each treatment by successive filtration of 2 L on 20 µm pore size nylon filter (Millipore, USA) and 0.65 µm pore size PVDF filter (Durapore, Germany) and stored at −80 °C.
3. To evaluate potential effects of the incubation conditions on plankton community composition, 2 × 5 mL of water were sampled at T_0_ and T_final_ from each carboy for cytometry analysis of pico-, and nano-phytoplankton abundances.
4. To determine more precisely the abundance of different taxonomic groups using FISH, 300 mL of water from the T_0_ carboy were filtered onto 0.4 µm polycarbonate filters (Nuclepore Track-Etch Membrane, Whatman, USA) and further dehydrated successively with 50, 70, and 100% ethanol for 3 min each [57] (Supplementary material).

### Preparation of samples for secondary ion mass spectrometry (SIMS)

Single-cell CO_2_-fixation analysis was performed with SIMS. For large cells, the 20 µm nylon filters were placed in 3 mL of 0.01 µm filtered seawater and gently vortexed to detach the cells. The solution was then pipetted onto a 0.2 μm polycarbonate membrane directly connected to a low-vacuum pump (<0.5 atmosphere) in order to concentrate the cells on a spot of about 2 mm^2^ on the filter. No samples were prepared for SIMS analysis of large cells at station M3 since almost no cells were collected on the 20 µm nylon filter during the sampling.

To detach and collect small cells (<20 µm), the 0.65 µm pore size PVDF filters were cut into small pieces, placed in a 3 mL solution composed of 0.01 µm filtered seawater and Kolliphor P188 (0.01% w/v final conc., Sigma-Aldrich), and sonicated twice for 1 min. Small autotrophs were then sorted using BD FACS Aria II flow cytometer (BD Biosciences, San Jose, CA, USA; UNICELL facility). Three different populations were gated at each station based on diverse combinations of red (690/50 nm, for chlorophyll *a* detection) and orange (585/42 nm, for phycoerythrin detection characteristic of *Synechococcus*) fluorescence, forward scatter (FSC, related to cell size) and side scatter (SSC, related to cell structure). *Synechococcus* cells were used as a standard to ensure homogeneity in the gating of cells of pico-size from one station to another. *Synechococcus* spp. abundances were low at all stations (45–400 cells mL^−1^), so that they were sorted together with picoeukaryotes (pigmented eukaryote cells in the same size range as *Synechococcus*) in a population hereafter called Pico (Fig. S2). Small pigmented nano-eukaryotes were sorted into two groups (Nano1 and Nano2) according to their red fluorescence and forward scatter. Sorted cells were directly collected onto a 0.6 μm polycarbonate membrane (DTTP01300, Millipore) in the sorting chamber using a low-vacuum in order to maximize cell density on the filter [27]. All filters were stored at −20 °C until analysis. Abundances of the populations sorted were determined in triplicates at the beginning and end of the incubations using a CytoFLEX (Beckman Coulter, Singapore) at a high flow rate (60 μl min^−1^) for 3 min. Homogeneity in the gating of the populations between the two flow cytometers used was ensured using *Synechococcus* as standard.

### SIMS analyses (Large geometry SIMS and nanoSIMS)

Pieces of the filters prepared for SIMS analyses were placed on double-sided conductive adhesive copper tape and mounted on plots adapted to SIMS samples holders. They were then metalized by sputter deposition of a gold film (20–50 nm thickness).

The ^13^C-fixation of large diatoms was measured using a large geometry SIMS (IMS1280, Cameca, Gennevilliers, France) at the Centre de Recherches Pétrographiques et Géochimiques (CRPG, CNRS-Univ. Lorraine, Nancy, France). Areas of interest (120 × 120 µm) were pre-sputtered with a primary 10 nA Cs^+^ beam for 5 min to remove the silica frustules of most diatoms and access their cellular content. Analyses were conducted on a 100 × 100 µm field using a 50–100 pA Cs^+^ beam with a spatial resolution of ~1.5 µm for 80 cycles. Secondary ion images (512 × 512 pixels) were recorded for ^12^C^14^N^−^ (2 s per cycle), ^13^C^14^N^−^ (4 s per cycle) and ^28^Si (2 s per cycle) at a mass resolution of 12 000 (M/ΔM).

The ^13^C-fixation of small pigmented cells sorted by flow cytometry in three populations (Pico, Nano1 and Nano2) was measured using a nanoSIMS 50 (Cameca, Gennevilliers, France) at the Museum National d’Histoire Naturelle (MNHN, Paris, France). NanoSIMS analyses were conducted on a field size of 40 × 40 µm (255 × 255 pixels) with a primary Cs^+^ ion beam of 1.2 pA with a lateral resolution of 60–120 nm for 1000 µs px^−1^. A larger field (42 × 42 µm) was pre-sputtered with a high primary ion beam current (300 pA) for 2–2.5 min. Secondary ions ^12^C, ^13^C, ^12^C^14^N^−^, ^13^C^14^N^−^, and ^28^Si were collected on at least 20 planes.

For large geometry SIMS and nanoSIMS images, regions of interest corresponding to single cells were manually defined using Limage software (Larry Nittler, Carnegie Institution of Washington) based on the total ^12^C^14^N^−^ ion counts. ^28^Si was further used to correct the shape of diatoms based on their silica frustule. The equivalent spherical diameter (ESD) was measured on nanoSIMS images and used to estimate biovolumes of small non-silicified cells. For small diatoms, the biovolume was calculated after Sun and Liu [58], taking measures on nanoSIMS images for each silicified cell. The average ESD of Pico, Nano1 and Nano2 cells were 1.6 ± 0.3, 2.5 ± 0.4, and 4.8 ± 1.6 µm, respectively.

A total of 344 cells were analyzed with large geometry SIMS and 2194 with nanoSIMS (1162 Pico, 944 Nano1, and 211 Nano2: Table S2). In addition, 774 non-enriched cells from the control carboys were analyzed to determine natural ^13^C isotopic content of phytoplankton cells.

### CO_2_-fixation calculations (EA-IRMS, large geometry SIMS, and nanoSIMS)

Bulk CO_2_-fixation rates measured by EA-IRMS (µmol C L^−1^ d^−1^) were calculated as follows:

$$Bulk\,{\mathrm{CO}}_2 {\hbox{-}} fixation \,=\, \frac{{A_{sample}^{POC} \,-\, A_{control}^{POC}}}{{A_{enriched}^{DIC} \,-\, A_{natural}^{DIC}}}xPOC_{sample}$$

Where A is the ^13^C isotopic fractional abundance (in atom%) of the community labeled with ^13^C after incubation $( {A_{{\mathrm{sample}}}^{{\mathrm{POC}}}} )$ of the T_0_ non-enriched samples $( {{\mathrm{A}}_{{\mathrm{control}}}^{{\mathrm{POC}}}} )$ of the enriched DIC source pool $( {A_{{\mathrm{enriched}}}^{{\mathrm{DIC}}}} )$ and of the natural DIC pool $( {A_{{\mathrm{natural}}}^{{\mathrm{DIC}}}} )$.

For each cell analyzed with nanoSIMS, ^13^C^14^N^−^ and ^12^C^14^N^−^ ions were counted and specific fractional abundance (A_Cell_) were calculated as follows:

$$A_{Cell} \,=\, \frac{{{\,}^{13}{\mathrm{C}}^{{\mathrm{14}}}N^ - _{(Cell)}}}{{{\,}^{13}{\mathrm{C}}^{{\mathrm{14}}}N^ - _{(Cell)} \,+\, {\,}^{12}{\mathrm{C}}^{{\mathrm{14}}}N^ - _{(Cell)}}} \,\times\, 100$$

To assess the metabolic activity of individual cells, C-based cell-specific division rates (d^−1^) were calculated as in Berthelot et al. [27], assuming that DIC was the only carbon source used for growth:

$$C {\hbox{-}} based\,cell {\hbox{-}} specific\,division\,rates(d^{ - 1}) \,=\, log_2\frac{{A_{DIC} \,-\, A_{Control}}}{{A_{DIC} \,-\, A_{Cell}}}$$

with *A*_control_ being the mean ^13^C cell fractional abundance in non-enriched populations. Cells whose fractional abundance enrichment *A*_Cell_ − *A*_Control_ was less than two times the standard deviation associated with the Poisson distribution parameterized by *λ* = *A*_Cell_ × *N*_CNcell_, with N_C__Ncell_ being the CN^−^ ion counts of the cell, were considered as inactive [27].

Contribution of the different population sorted by flow cytometry was calculated by multiplying the mean cell-specific CO_2_-fixation by the abundance of the population.

For this, the C-based turnover of the cellular C-content was calculated as follows:

$$C {\hbox{-}} based\,turnover\,of\,the\,cellular\,C {\hbox{-}} content = \frac{{A_{Cell} - A_{Control}}}{{A_{DIC} - A_{Control}}}$$

Cell-specific CO_2_-fixation (fmol C cell^−1^ d^−1^) were obtained by multiplying the C-based turnover of the cellular C-content by the carbon content of the cell, calculated after Verity et al. [59]:

$$C {\hbox{-}} cell\,content \,=\, 0.433 \,\times\, Biovolume^{0.863}$$

### Statistical analysis

All statistical analyses were conducted in R [60]. Differences in CO_2_-fixation rates between groups and stations were assessed using the Kruskal–Wallis test, followed by pairwise Mann–Whitney test with Bonferroni correction for multiple comparisons with ggpubr package. Interquartile range (IQR) was used as a measure of statistical dispersion within groups. The package fitdistrplus was used to select the best probability distribution fitting the division rates observed for small and large cells.

## Results

### Study area

Chl *a* was low at all stations and visits (0.18–0.31 and 0.28–0.58 µg L^−1^ on- and off-plateau, respectively). Contrasted nutrient concentrations were observed on- and off-plateau (Table 1). Plateau station M2 was depleted in silicic acid (<2 µmol L^−1^), but silicic acid concentrations and Chl *a* doubled at the last visit (M2-3) after a storm on the 10th March. Ammonium concentrations were higher at M2 than at off-plateau stations. Off-plateau stations sampled in HNLC waters presented higher nitrate, silicic acid, and phosphate concentrations than on-plateau (Table 1). Stations M1 and M4, south of the polar front, were characterized by lower temperature and higher silicic acid concentrations than M3, located in subantarctic waters north of the polar front (Fig. 1).

### Composition of phytoplankton communities

**Fig. 2:**
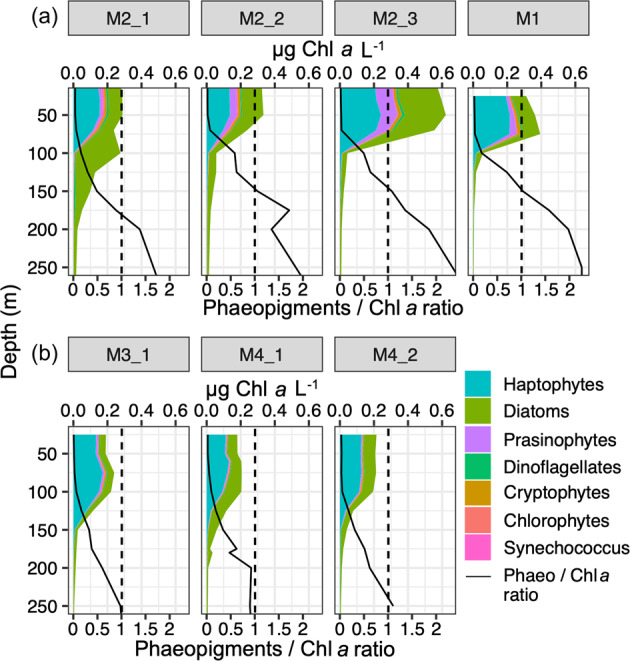
Contribution of major phytoplankton groups to total Chl a with depth. Total Chl *a* concentrations correspond to the cumulative concentration of taxon-specific Chl *a*. The panels are organized here according to the rate of decrease of small phytoplankton pigments. The black line indicates Phaeopigment:Chl *a* ratio. Phaeopigments correspond to degraded and Chl *a* to fresh pigment material. The dashed black line corresponds to a ratio of 1. **a** Stations have Phaeo/Chl *a* ratio above 1 at 200 m depth, whereas **b** stations the ratio is <1.

Haptophytes and diatoms contributed the most to Chl *a* at the surface at all stations based on CHEMTAX analysis (36–70% and 18–40%, respectively: Fig. 2a, b). Chl *a* concentration strongly decreased between 75 and 125 m depending on the station. Down to 250 m, Chl a concentrations were low (0.01 µg Chl *a* L^−1^) and diatoms accounted for 77–96% of total Chl *a*.

Vertical distribution of haptophyte pigments and the Phaeo/Chl *a* ratio differed between stations (Fig. 2a, b). At M2 and M1 stations, haptophyte pigments were abundant at the surface but decreased rapidly with depth and almost disappeared below 75 m. These stations were also characterized by Phaeo/Chl *a* ratios >1 below 175 m, indicating that pigments found below this depth were mostly degraded. At off-plateau stations M3 and M4, haptophyte pigment signatures extended deeper. Phaeo/Chl *a* ratio was approximately equal to 1 at 250 m, reflecting a similar contribution of fresh and degraded pigments at this depth (Fig. 2b).

**Fig. 3:**
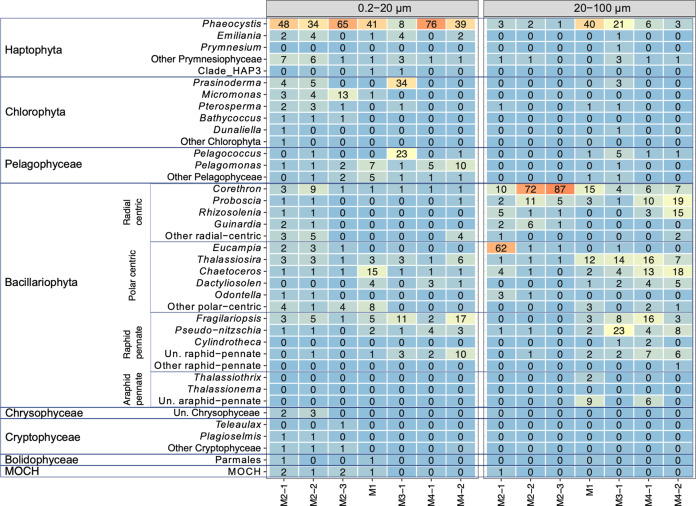
Heatmap showing relative abundances of sequence reads for surface phytoplankton taxa in the small (0.2–20 µm) and large (20–100 µm) size fractions. Taxa are grouped by division (Haptophyta, Chlorophyta) or class (Bacillariophyta, Pelagophyceae, Dinophyceae, Chrysophyceae, Cryptophyceae, Bolidophyceae, and MOCH).

Sequencing data revealed that *Phaeocystis antarctica* (haptophyte) was the most abundant phytoplankton taxa in the small size fraction on- and off-plateau (up to 76% of the reads: Fig. 3). Other common non-silicified phytoplankton taxa of the small size fraction included chlorophytes *Prasinoderma* (Prasinococcales family, 34% of the reads at M3) and *Micromonas* (Mamiellaceae family, 3–13% of the reads at M2). CARD-FISH counts confirmed the importance of haptophytes (2–5 µm in size) on- and off-plateau (735–4950 cells mL^−1^), and of prasinophytes (<2 µm in size) on-plateau (Fig. S3). Members of the Pelagophyceae family were common at off-plateau stations, in particular *Pelagococcus* (23% of the reads at M3) and *Pelagomonas* (5–10% at M4 and M1, respectively). Diatoms contributed for 10–45% of the total number of reads in the small size fraction, with a higher contribution of raphid pennates (*Fragilariopsis* and unidentified raphid pennates) off- than on-plateau (8–30% and 4–6% of reads number, respectively: Fig. 3).

Diatoms were the dominant phytoplankton class of the large size fraction (>20 µm in size; 55–97% of the reads). Microscopic observations confirmed that the size range of several diatom genera overlapped the two size fractions [61]. Off-plateau, diatom communities were composed of pennate (*Fragilariopsis*, *Pseudo-nitzschia*) and centric diatoms (*Thalassiosira, Chaetoceros, Proboscia*, and *Rhizosolenia*). On-plateau, large diatom communities were dominated by centric diatoms (*Eucampia* during the first visit and *Corethron* for the two last visits). *Phaeocystis* was abundant in read numbers in the large size fraction at M1 and M3−1 (40 and 21% of the reads, respectively).

### C-based division rates of small and large cells

**Fig. 4:**
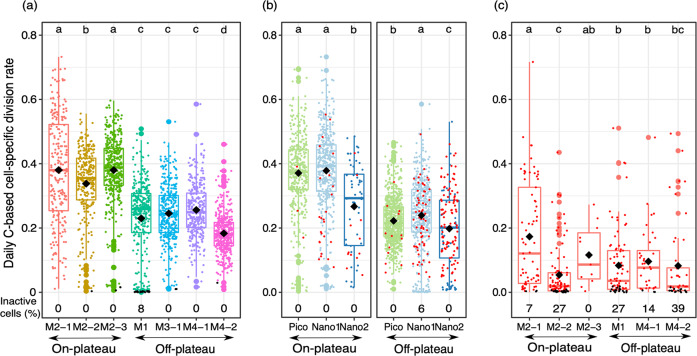
Boxplot of the daily CO_2_-based cell-specific division rates. Each dot corresponds to the division rate of a single-cell measured with NanoSIMS for cells <20 µm (**a**, **b**) or with SIMS for diatoms >20 µm (**c**). Diamonds indicate mean division rates and inactive cells are colored in black. Significant differences (pairwise Mann–Whitney test with *p* < 0.05) in division rates between stations (**a**) or between size-groups on- and off-plateau (**b**) are indicated by letters above the boxplots (ranked by alphabetical order from highest to lowest division rates). Outliers correspond to the larger points.

Over 98% of the small cells measured with nanoSIMS were actively taking up carbon, with the exception of M1 where slightly less cells were active (92%: Fig. 4a). Division rates were significantly higher on- than off-plateau (mean from 0.33–0.38 and 0.18–0.26 division d^−1^, respectively: Fig. 4a). Division rates were similar at the three off-plateau stations, no matter their position on the polar front (Fig. 4a). However, Nano2 cells were characterized by lower division rates than Pico and Nano1 on- and off-plateau (Fig. 4b). Nano2 also presented higher variability and higher IQR of the division rates than the two other small cells groups. Interestingly, Nano2 was mostly composed of small diatoms off-plateau (70%), while non-silicified cells were the major contributors of this group on-plateau (81%). Division rates of small diatoms were significantly lower than those of non-silicified cells on-plateau (Mann–Whitney, *P* < 10^−9^), but they were not different off-plateau (Fig. S4). Division rates of small cells (Pico, Nano1, and Nano2) followed a symmetrical logistic distribution, very similar to the normal distribution (Fig. S5a, b).

The mean division rates of larger diatoms (>20 µm in size) were relatively low and showed great variability (0.11 ± 0.14 and 0.09 ± 0.11 division d^−1^). Mean division rates of large diatoms on-plateau were 0.17, 0.05, and 0.12 division d^−1^ during the first, second, and third visit at M2, respectively, while they ranged between 0.08 and 0.10 division d^−1^ at off-plateau stations (Fig. 4c). The proportion of inactive diatoms varied from 0 to 27% and 14 to 39% on- and off-plateau, respectively. However, some active outliers (<7% of the diatoms measured with large geometry SIMS) showed high division rates reaching 0.72 and 0.51 division d^−1^ on- and off-plateau, respectively (Fig. 4c). As a consequence, the distribution best fitted to large diatoms’ division rates was a log-normal distribution skewed towards low values (Fig. S5c).

### CO_2_-fixation by small phytoplankton

**Fig. 5:**
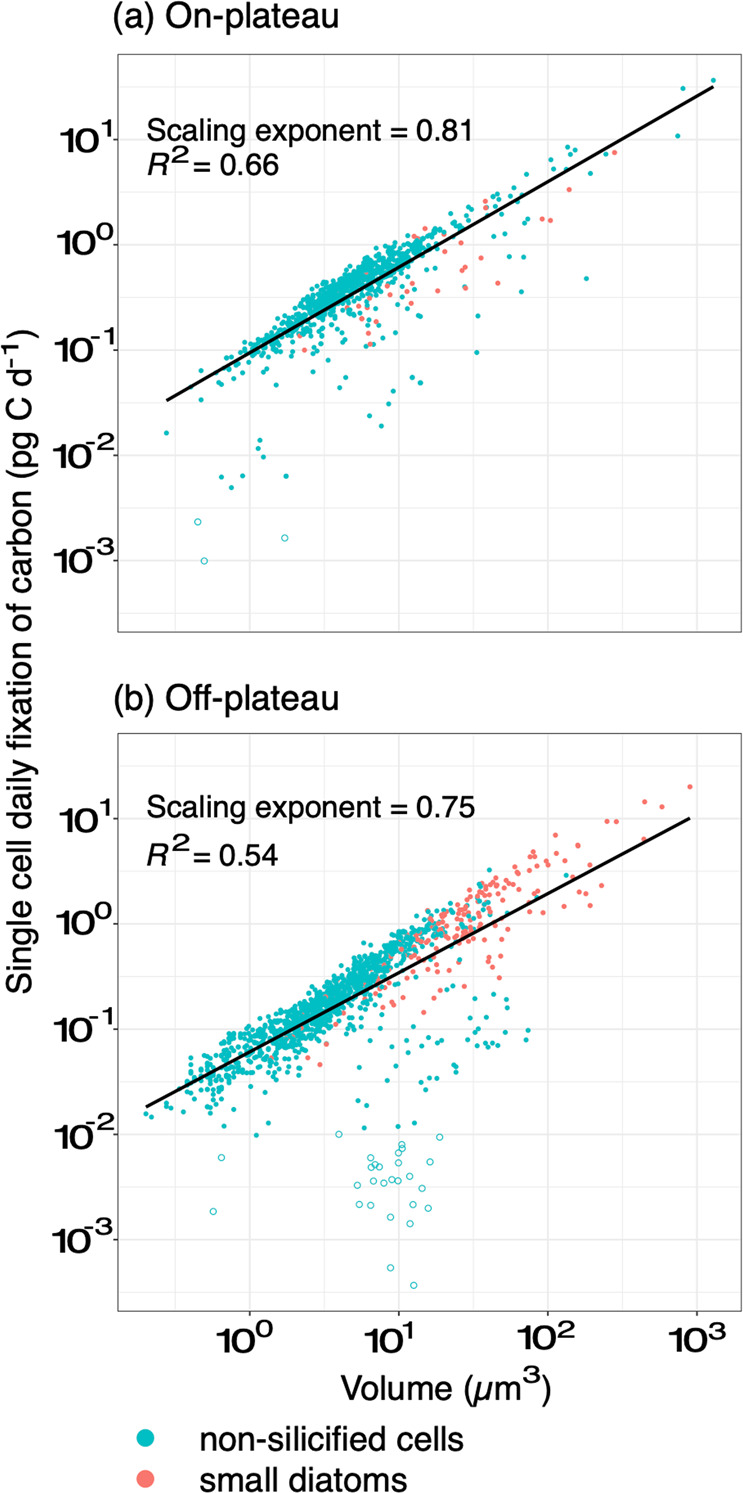
Relationship between single-cell daily uptake rates of carbon (pgC d^−1^) and single-cell volumes for small diatoms and nonsilicified cells (<20 µm) on- (a) and off-plateau (b). Empty circles correspond to inactive cells. Scaling exponents have been obtained by linear least-squares fitting of log-transformed data. Consequently, the amount of CO_2_ fixed at the single-cell level (C-fix) scaled with cell volume (V) according to the power law C-fix = aV^α^ where a is a constant that differed on- and off-plateau and α is the scaling exponent.

For small cells, the amount of carbon fixed at the single-cell level (C-fix) scaled allometrically with cell volume (V) according to a power law C-fix = aV^α^, where the scaling exponent α = 0.81 and 0.75 on- and off-plateau, respectively (Fig. 5). This relationship explained 66% of the variance observed in CO_2_-fixation of individual cells on-plateau and 54% off-plateau, where a few inactive cells departed from this relationship. Mean daily CO_2_-fixation rates at each station were highest for Nano2, intermediate for Nano1 and the lowest for Pico-cells (Table S3). However, when normalized to cell volume, the volume-specific CO_2_-fixation rates were decreasing with size (Fig. S6).

**Fig. 6:**
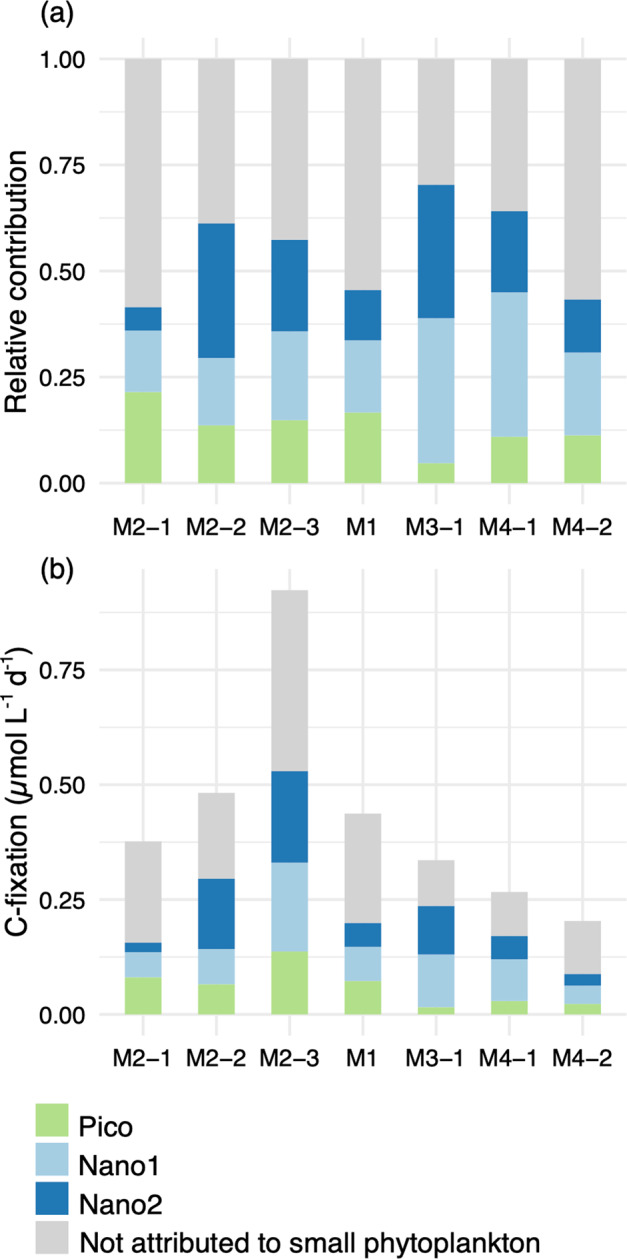
Contribution of small phytoplankton to bulk CO_2_-fixation. Relative (**a**) and absolute (**b**) contribution of the different groups of small phytoplankton to bulk CO_2_-fixation were obtained by multiplying mean CO_2_-fixation rates (nanoSIMS) by the abundance of the groups (flow cytometry enumeration). Bulk contribution was measured with EA-IRMS.

Estimated contribution of the different small phytoplankton’s size-groups to total CO_2_-fixation was important on- and off-plateau (41–61% and 43–70% on- and off-plateau, respectively; Fig. 6a). Nano1 was the most important contributor within small autotrophs to CO_2_-fixation at all stations (17–34%) except M2-1 where Pico contribution was higher (21%). Total community CO_2_-fixation off-plateau varied between 0.20 and 0.44 µmol C L^−1^ d^−1^(Fig. 6b). The CO_2_-fixation on-plateau was slightly higher during the first two visits (0.37–0.48 µmol C L^−1^ d^−1^) and doubled at the third visit (0.92 µmol C L^−1^ d^−1^; Fig. 6b). The doubling of the CO_2_-fixation at the last visit at M2 was associated with the doubling of Chl *a* concentration as well as increases in abundances of the three small phytoplankton size-groups (Fig. S3; Table S2). Estimation of the contribution to CO_2_-fixation of large diatoms by extrapolation of their CO_2_-fixation rates was not possible because of: (1) the low number of large diatoms analyzed; and (2) the variability observed in their division rates in post-bloom conditions, with mean division rates very sensitive to the presence of active outliers. However, large diatoms probably account for most of the CO_2_-fixation not attributed to small phytoplankton.

## Discussion

We report here, for the first time, that small phytoplankton (mainly non-silicified) could represent 41–61% of the total CO_2_-fixation in post-bloom conditions on the Kerguelen Plateau, a naturally iron-fertilized area previously characterized by the dominance of chain-forming and large diatoms. Previous estimates of small phytoplankton contribution to CO_2_-fixation in other naturally iron-fertilized regions of the SO were usually much lower (Table 2). This high contribution on- and off-plateau was achieved by different communities of small phytoplankton, mostly represented by non-silicified pico and nano-eukaryotes on-plateau, whereas small diatoms (3.8 ± 1.5 µm ESD; Fig. S7) were also abundant and active off-plateau (Fig. 4b). Complementary SIMS analysis revealed that many larger diatoms (>20 µm) were inactive at this time of the season and that most of the CO_2_-fixation within this group was achieved by a few cells only (Fig. 4c).

**Table 2:** Contribution of small phytoplankton to CO_2_-fixation on and off iron-fertilized areas of the Southern Ocean.

Study area (experiment name)	Type of Fe-fertilization	Small phytoplankton size (µm)	HNLC (%)	Fe-fertilized (%)	Month	Method	Source
Crozet (CROZEX)	Natural Fe	<20	66	53	November - January	Size-fractionation	[83]
Kerguelen (KEOPS1)	Natural Fe	<10	68	10–20	February	Size-fractionation	[12]
Amundsen Sea	Natural Fe	<5	51	15	January	Size-fractionation	[84]
South Georgia	Natural Fe	<12	>60	<20	January	Size-fractionation	[85]
South of Australia (SOIREE)	Artificial	<20	>60	Decrease to <40 during the experiment	February	Size-fractionation	[86]
Atlantic sector of the so (EISENEX)	Artificial	<20	70–90	Decrease to <50 during the experiment	November	Size-fractionation	[87]
Kerguelen (MOBYDICK)	Natural Fe	<20	43–70	41–61	March	NanoSIMS	This study

During artificial fertilization studies, the initial contribution of small phytoplankton corresponds to the HNLC value. This contribution decreased throughout the fertilization experiments as the contribution of larger phytoplankton increased.

### Drivers of small phytoplankton importance in CO_2_-fixation in contrasted areas

In this study, the contribution of the three size-groups of small cells to bulk CO_2_-fixation was comparable on- and off-plateau (41–70%; Fig. 6a). As C-based division rates of small cells differed on- and off-plateau (Fig. 4a, b), different mechanisms may explain the importance of small cells in CO_2_-fixation in these two areas after the bloom. In HNLC waters, high contribution of small phytoplankton to CO_2_-fixation is a commonly observed phenomenon (Table 2), attributed to the advantage of a reduced size in iron acquisition [62–64]. In our study, smaller cells showed higher volume-specific CO_2_-fixation rates than their larger counterparts, in line with their theoretical advantage of high surface/volume ratio for nutrient and light uptake. This theoretical allometric relationship has not always been verified, as some studies have suggested that CO_2_-fixation could also scale isometrically with cell volume, and that larger cells could be as, or even more competitive, than smaller ones depending on the environmental conditions [65, 66]. During MOBYDICK, an allometric size-scaling relationship was observed for the three small cell-size groups. This relationship explained over half of the variability observed in CO_2_-fixation of small phytoplankton cells ranging over four orders of magnitude (66% on- and 54% off-plateau: Fig. 5). Other sources of variability in CO_2_-fixation may come from taxa-specific physiology adapted to on- and off-plateau conditions. For example, pelagophytes and small pennate diatoms were mostly present off-plateau and *Micromonas* on-plateau (Fig. 3). The lower C-based division rates observed at off-plateau stations (Fig. 4a) likely resulted from higher iron limitation, whereas iron is continuously supplied to surface waters by internal waves on-plateau [38]. Higher competitiveness with respect to iron acquisition may favor pelagophytes and pennate diatoms off-plateau. Hogle et al. [67] observed over-expression of genes involved in iron metabolism in a metatranscriptomic study, suggesting pelagophytes were advantaged in HNLC waters. As for pennate diatoms, they possess the iron storage protein ferritin, which enables them to store iron on the long term and to be very efficient in using pulsed iron inputs [68, 69]. On-plateau, higher ammonium and lower silicic acid concentrations were observed than off-plateau. The relatively high ammonium concentrations could have benefited to the growing *Micromonas* population (Figs. 3 and S3), since prasinophytes preference for ammonium could be tenfold superior to other phytoplankton groups [70]. In contrast, silicic acid limitation could have limited small diatom’s growth on-plateau in comparison to small non-silicified cells after the bloom (Fig. S4) and explain why fewer small diatoms were observed on- than off-plateau (Fig. 4b). Finally, some of the variability observed in CO_2_-fixation of small cells may originate from physiological heterogeneity within a species. For example, *P. antarctica* which was the most abundant taxa on- and off-plateau is characterized by highly variable responses to iron limitation, even within clonal populations (i.e., size reduction, decrease of Chl *a* concentration; [71]).

Currently, little information is available on in situ division rates of small phytoplankton taxa in the SO, most of them been obtained from *Phaeocystis* cultures (Table S4). Despite the variability observed at the single-cell level, mean division rates observed in our study on- and off-plateau were in the same range as the ones observed for *P. antarctica* in Fe-replete and Fe-limited cultures. Therefore, we suggest that the division rates measured in this study in natural communities composed of diverse phylogenetical groups could serve as a baseline to model small phytoplankton growth after the bloom in HNLC (mean of 0.22 ± 0.09 division d^−1^) and naturally iron-fertilized areas (0.37 ± 0.13 division d^−1^).

On-plateau, the increasing contribution of small cells to bulk CO_2_-fixation after the bloom mainly resulted from the senescence of larger diatoms in post-bloom conditions. Many large diatoms (>20 µm) were not actively growing, while few cells showed high CO_2_-fixation (Fig. 4c). Most likely, division rates of large diatoms change considerably throughout the season in relation with silicic acid and iron availability. Silicic acid concentrations on-plateau can be as high as 19 µmol L^−1^ in early spring at the onset of the bloom [72]. After the bloom, silicic acid concentrations were <2 µmol L^−1^ during the first two visits at M2, a level which is considered as an empirical threshold to support diatoms’ dominance over flagellates [73]. Off-plateau, large diatoms are likely primarily limited by iron. The high proportion of inactive large diatoms observed with SIMS was in line with microscopic observations of surface samples during MOBYDICK showing 33 ± 7 % of empty/broken frustules [61]. It is worthy to note that highly heterogeneous division rates have been observed in culture studies within large diatoms (Table S4) in Fe-limited cultures (e.g., daily division rates from 0.03 to 0.43 d^−1^) and also within specific genera in Fe-replete conditions (e.g., daily division rates from 0.16 to 0.64 d^−1^ for *Fragilariopsis* sp.). These intriguing results relative to the highly heterogeneous division rates of larger diatoms observed in our study highlight the need to further explore species-specific changes in CO_2_-fixation rates at the single-cell level in response to contrasted environmental conditions.

### Indications on the fate of small phytoplankton

Currently, export fluxes in the SO cannot be predicted based on global primary production and food web structure. Several studies conducted in the SO have revealed an inverse relationship between primary production and carbon export efficiency [74–76]. This decoupling between the carbon produced in the surface layer and the carbon export efficiency below 200 m has also been documented on the Kerguelen Plateau, where high productivity regime during early spring was associated with low carbon export efficiency (1–2%), and moderate productivity in summer showed high export efficiency (26%; [77, 78]). In low-productive HNLC waters of the Kerguelen Plateau, high carbon export efficiencies were observed in spring and summer (35% and 44%, respectively; [77, 79]). Although the factors driving this inverse relationship between primary productivity and export efficiency are not fully understood, micro- and macro-zooplankton-mediated grazing seem to be an efficient alternative pathway to export carbon in low productivity waters [76, 80]. Counter to the classical view that only large phytoplankton are exported due to their high sinking velocity [8], there is growing evidence that the relative contribution of small phytoplankton to total C-export is proportional to its contribution to total primary productivity, when indirect export pathways (such as grazing through the production of fecal pellets by higher trophic levels) were also considered [11]. Considering the important contribution of actively growing small cells to CO_2_-fixation in the surface layer in our study, their possible export pathways—in particular indirectly via grazing—deserve some attention. Interesting observations relative to grazing could shed light on the vertical pigment distribution observed during MOBYDICK where pigments of small non-silicified groups (haptophytes and prasinophytes mostly) were almost absent below 100 m.

Grazing measurements showed that microzooplankton grazed actively on phytoplankton at all stations with grazing rates exceeding phytoplankton growth rates (Christaki et al., submitted). Consequently, an important part of the carbon fixed by small phytoplankton at the surface may have been assimilated by microzooplankton and channeled to higher trophic levels. The Phaeo/Chl *a* ratio showed that grazing activity was intensified at stations M2 and M1 (Fig. 2a). These stations were characterized by higher productivity in the months before sampling. The bloom ended ~1 month before MOBYDICK [81], so that part of the higher Phaeo/Chl *a* ratio observed at these stations could still reflect past grazing activity during the bloom. These two stations were also characterized by dense salps populations (*Salpa thompsoni*), making up 41–42 % of total micronekton biomass while they were almost absent at M3 and M4 [82]. Salps are major grazers of small phytoplankton in the SO [83] and produce easily fragmented fecal pellets in the upper mesopelagic layer [84], which could explain the pronounced Phaeo/Chl a ratio below the mixed layer at M2 and M1. Finally, molecular analysis of plankton communities at 300 m revealed that 25% of the sequences recovered in HNLC waters in the >20 µm size fraction belonged to *P. antarctica*, confirming the contribution of small phytoplankton to carbon export through fecal pellet export, direct sinking of ungrazed large colonies and/or aggregation in low-productive waters [85–87]. Our observations underline that grazing and aggregation may be important pathways of small phytoplankton export in both productive and HNLC waters.

Concluding, this study has shown for the first time the importance of actively growing small (silicified and non-silicified) phytoplankton cells in iron-fertilized and HNLC waters of the SO during post-bloom conditions, when large diatoms were decaying. Single-cell analysis revealed higher homogeneity in CO_2_-fixation within small phytoplankton composed of diverse phylogenetically distant taxa (prymnesiophytes, prasinophytes, and small diatoms) than within large diatoms which were likely limited by silicic acid and iron in post-bloom conditions. Considering the high inter-annual variability and limited duration (~4 months) of diatom blooms, our data highlight the need to reassess the role of small phytoplankton in the SO when large diatoms growth is limited by bottom-up processes. Further investigation of the indirect contribution of small phytoplankton to C-export via grazing is also needed as it may be an efficient export pathway especially in HNLC waters characterized by sparse productivity pulses. Data of phytoplankton division and CO_2_-fixation rates published here will also be useful for modeling parameterization of phytoplankton size-group contribution to the C-cycle in the SO.

## Supplementary information

Supplementary figures and tables
