# Supplementary material for: Small phytoplankton contribute greatly to CO2-fixation after the diatom bloom in the Southern Ocean
Source: ISME J. 2021 Mar 12;15(9):2509–22. doi: 10.1038/s41396-021-00915-z (PMC8397732; doi:10.1038/s41396-021-00915-z)
Supplement: Supplementary file 1 — Supplementary figures and tables [file 41396_2021_915_MOESM1_ESM.pdf]

Supplementary material

Catalyzed reporter deposition of Fluorescent In Situ Hybridization (CARD-FISH)

Fluorescent in situ hybridization with horseradish peroxidase-labelled probes was performed as detailed in Not et al. [1] to quantify the abundances of the major phytoplankton phylogenetic groups. Diverse probes (EUK1209r, NCHLO01 and CHLO02) were combined to target all eukaryotes and a set of probes (PRAS04, PRYM02 and PELA01) were used separately to target most of the prasinophytes, prymnesiophytes, and pelagophytes [1–3]. In brief, filter pieces were incubated in the hybridization buffer (2 µl of oligonucleotide probe at 50 ng μl^{-1} stock, 40% formamide, 0.9 M NaCl, 20 mM pH 7.5 Tris-HCl, 0.01% (w/v) sodium dodecyl sulphate (SDS) - Sigma-Aldrich) and incubated at 35°C for 3 h. Filters were then washed twice at 37°C for 20 min with a washing buffer (56 mM NaCl, 5 mM EDTA, 0.01% (w/v) SDS, 20 mM Tris-HCl, pH 7.5) and equilibrated at room temperature for 15 min in TNT buffer (100 mM Tris-HCl, pH 7.5, 150 mM NaCl, 0.05% (v/v) Tween 20 - Sigma-Aldrich). TSA (fluorescein Tyramide reagent pack - Perkin Elmer) was performed in 20 µl of TSA mix following manufacturer's recommendations for 30 min in the dark at room temperature. Finally, filters were transferred into a TNT buffer and incubated twice at 55°C for 20 minutes. Filters were then mounted in the anti-fading reagent AF1 (Citifluor, London, UK) mixed with propidium iodide (1 µg mL^{-1} final conc.). Samples were then stored at -20°C until analysis (<1 week) with a Zeiss imager M2 epifluorescence microscope.

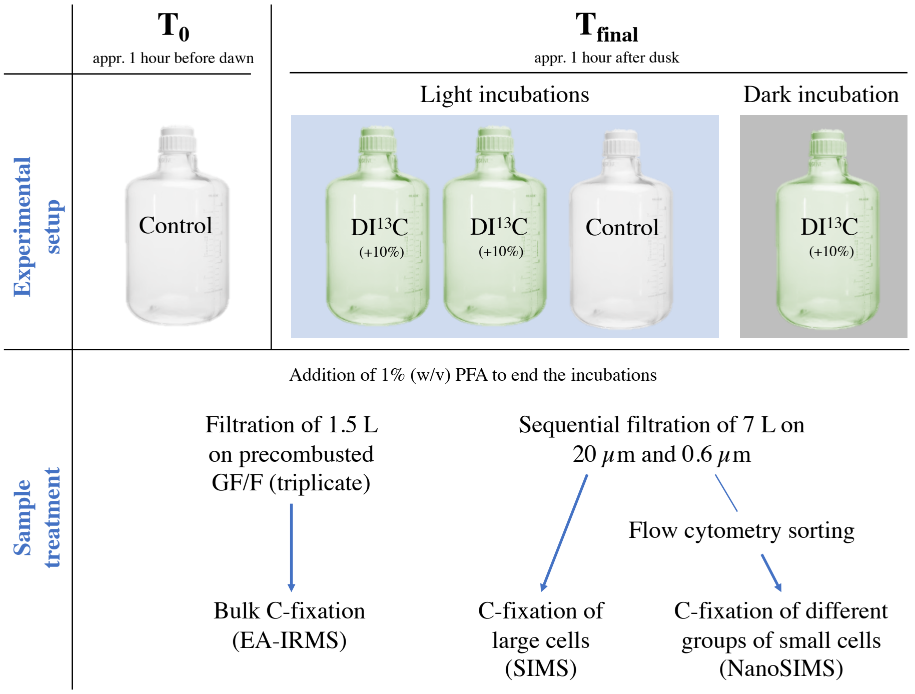

Fig. S1: Experimental setup of the study.

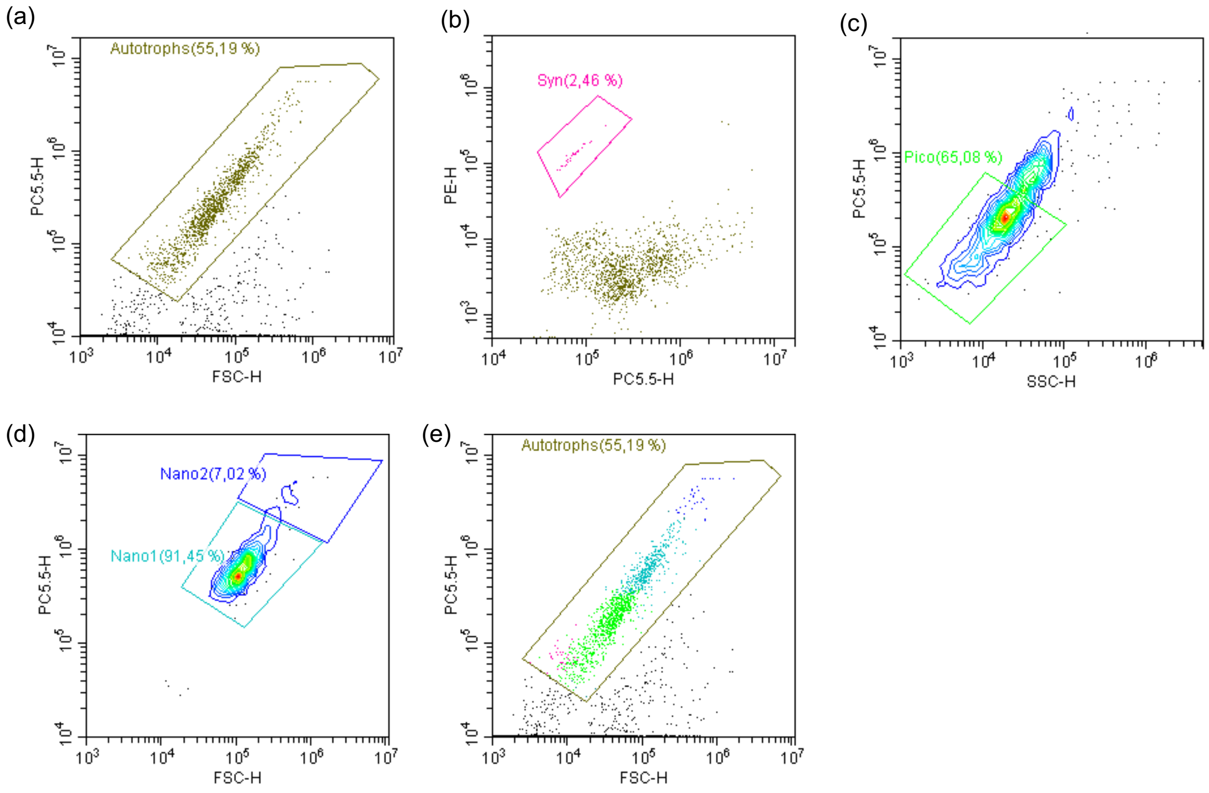

Fig. S2: Step by step protocol for the gating and sorting of small phytoplankton groups. Pigmented cells were first gated based on their chlorophyll (PC5.5-H) and forward scatter (FSC-H) (a). Only pigmented cells were plotted and *Synechococcus* cells gated based on their higher phycoerythrin signal (PE-H) (b). Pico group was created to group *Synechococcus* cells with pico-eukaryotes in the same size range (c). The rest of the autotrophs were separated in two groups (Nano1 and Nano2) based on their FSC-H (d). All groups were plotted based on their Chl *a* and forward scatter signal (Syn and pico-eukaryotes in pink and light green; Nano1 in turquoise and Nano2 in dark blue) (e).

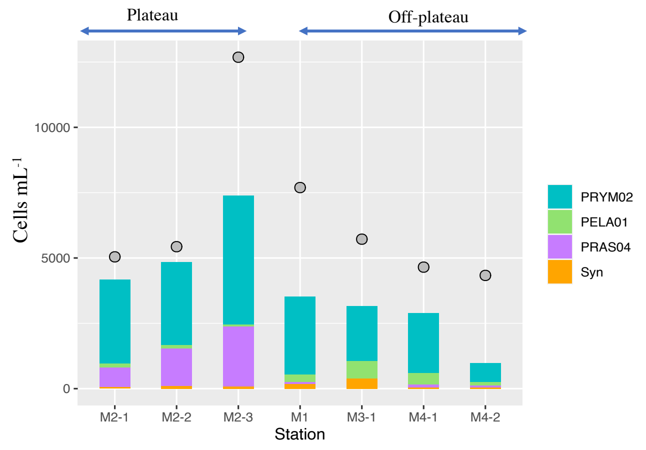

Fig. S3: Surface abundance of different groups of small non-silicified autotrophs assessed by CARD-FISH labeling with probes PRAS04 (prasinophytes, Mamiellaceae family), PRYM02 (haptophytes) and PELA01 (pelagophytes). Abundance of *Synechococcus* obtained by flow cytometry is also indicated (Syn). Total abundances of small autotrophs enumerated by flow cytometry are indicated at the top of each column with a grey dot. Counts with the CARD-FISH method confirmed the importance of haptophytes (2-5µm in size) on- and off-plateau (735 – 4950 cells mL-1 depending on the station). Prasinophytes (<2µm in size) from the Mamiellaceae family were more abundant on-plateau (750-2300 and 70-115 cells mL-1 respectively), while pelagophytes (appr. 3 µm in size) were mostly observed off-plateau. These three groups accounted for 58-87% and 22-61% of small phytoplankton abundances enumerated by flow cytometry on- and off-plateau, respectively.

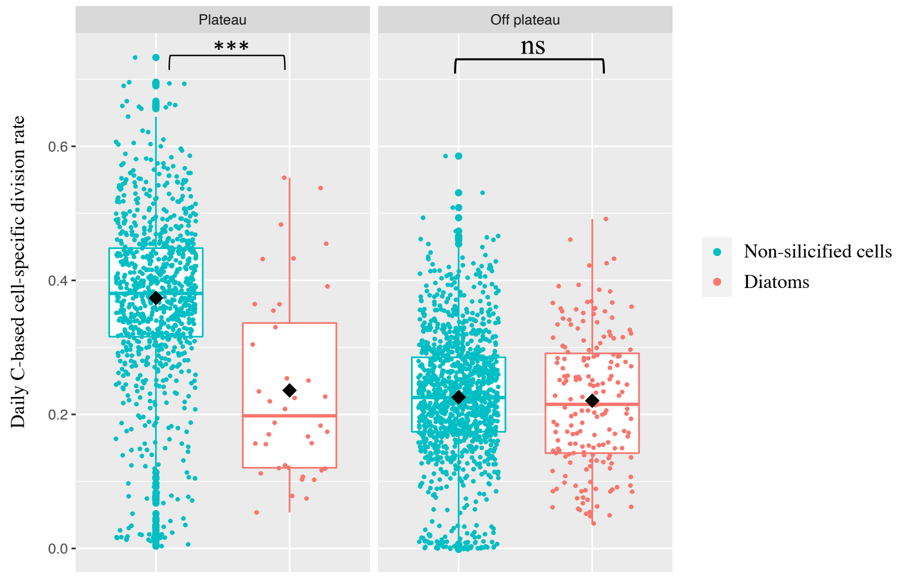

Fig. S4: C-based cell-specific division rates measured with nanoSIMS for small diatoms and non-silicified cells on- and off-plateau. Diatoms' division rates were significantly lower than those of non-silicified cells on-plateau (Kruskal-Wallis, $P<10^{-8}$), but not off-plateau. Note the low number of small diatoms on-plateau, suggesting their limitation by silicic acid.

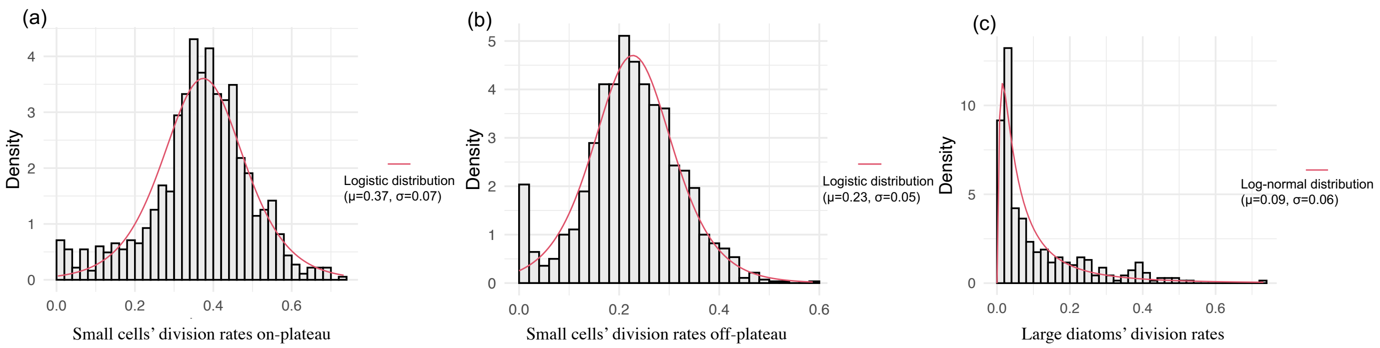

Fig. S5: Histogram of measured division rates of small cells on (a) and off- plateau (b) and large diatoms (c). The best distribution was selected from a predefined family of distributions (normal, logistic, exponential, log-normal, uniform, gamma) based on the lowest AIC. Division rates of small cells were best fitted by a logistic distribution on and off-plateau with mean division rates μ=0.37 and μ=0.23 on- and off-plateau respectively. Division rates of large diatoms fitted a log-normal distribution characterized by skewed distributions to the left (low division rates) with mean division rates μ=0.09.

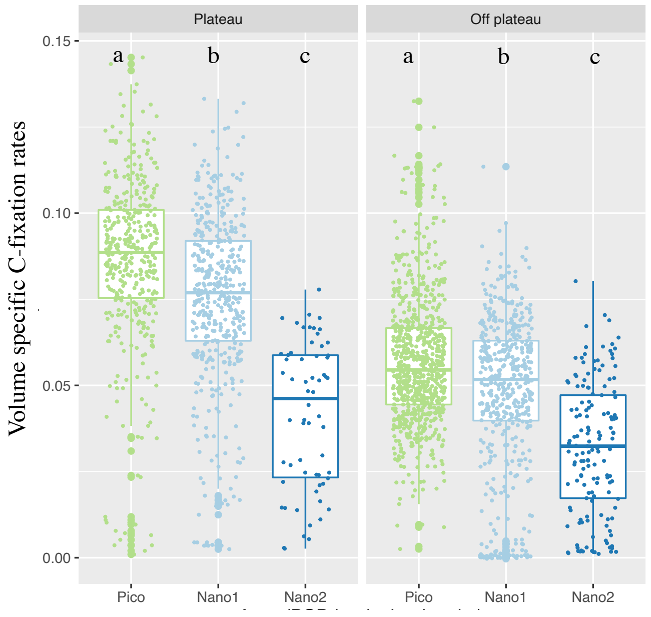

Fig. S6: Single-cell volume-specific C-fixation rates (i.e. the daily carbon fixation rate expressed in fg C divided by the cell volume). Significant differences are indicated by letters above the boxplots ranked by alphabetical order from highest to lowest mean volume-specific C-fixation rates (pairwise Mann-Whitney test with P<0.05)

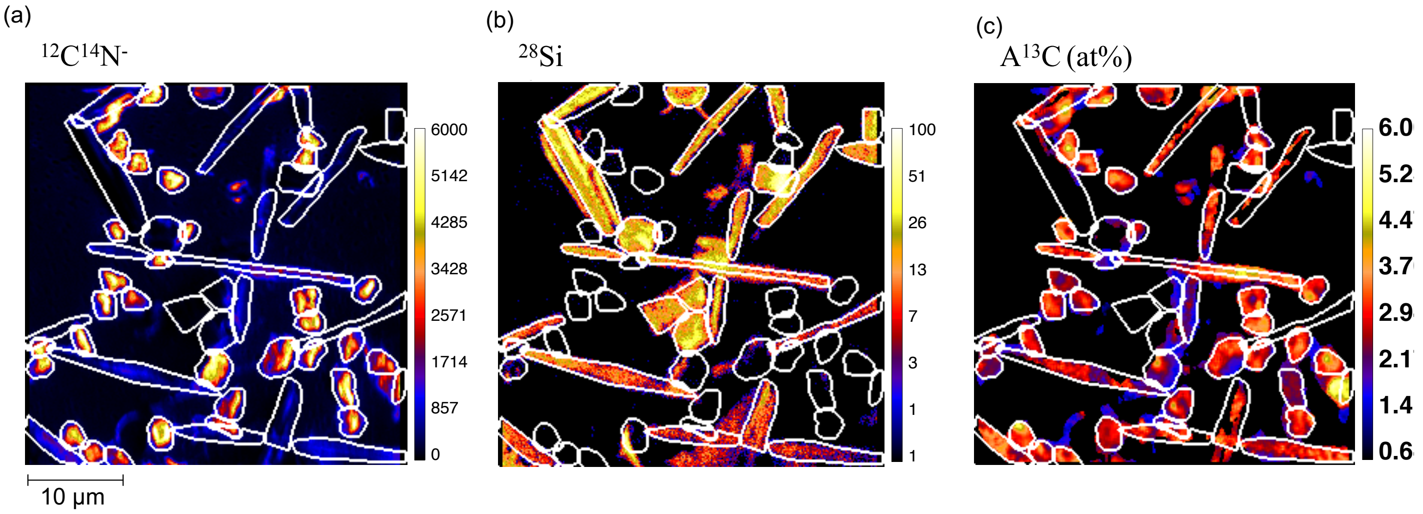

Fig. S7: Examples of nanoSIMS images showing the sum of $^{12}C^{14}N^-$ (a) and ^{28}Si ions detected (b), as well as the $A^{13}C$ enrichment (c) for Nano1 at M4-1. The frustules of small diatoms are colored in (b).

Table S1: Summary of the ^{13}C fixation incubations' location and timing.

	Station	Longitude (degrees east)	Latitude (degrees north)	Incubation date	Start time	End time
Off-plateau	M1	74.90	-49.85	09/03/2018	03:20	20:15
	M3_1	68.06	-50.68	05/03/2018	05:40	20:15
	M4_1	67.20	-52.60	01/03/2018	05:20	20:10
	M4_2	67.20	-52.60	12/03/2018	04:30	20:15
Plateau	M2_1	72.00	-50.62	26/02/2018	05:20	20:15
	M2_2	72.00	-50.62	07/03/2018	05:10	20:30
	M2_3	71.99	-50.62	17/03/2018	03:40	19:55

1

Table S2: Details of the cells analyzed with nanoSIMS and SIMS.

	Population	Station	Abundance (cells mL-1)	Cells analyzed	Diatoms analyzed	% diatoms	Nb of inactive cells	% Inactive cells	Mean diameter (µm +/- sd)	Mean division rate	Median division rate	Interquartile range (IQR)
NanoSIMS	Pico		3517	69	2	3%	-	-	1.78±0.16	0.41	0.43	0.21
	Nano1		1190	153	16	10%	-	-	2.43±0.3	0.37	0.35	0.27
	Nano2	M2-1	337	3	3	100%	-	-	3.71±0.04	0.15	0.12	0.05
	Pico		3410	96	-	-	1	1%	1.76±0.16	0.34	0.35	0.14
	Nano1		1603	145	9	6%	-	-	2.48±0.36	0.36	0.37	0.10
	Nano2	M2-2	420	33	9	27%	-	-	5.62±2.03	0.25	0.28	0.24
	Pico		8153	237	1	0%	2	1%	1.58±0.28	0.37	0.37	0.10
	Nano1		3753	155	-	-	-	-	2.43±0.35	0.41	0.43	0.11
	Nano2	M2-3	777	26	-	-	-	-	4.63±1.83	0.30	0.34	0.15
	Pico		5090	123	3	2%	-	-	1.72±0.17	0.26	0.26	0.05
	Nano1		2280	167	17	10%	29	17%	2.52±0.38	0.24	0.28	0.19
	Nano2	M1	325	59	29	49%	-	-	4.8±1.74	0.14	0.12	0.21
	Pico		2260	245	1	0%	2	1%	1.3±0.31	0.25	0.25	0.12
	Nano1		2830	85	4	5%	-	-	2.61±0.45	0.23	0.23	0.10
	Nano2	M3-1	630	20	13	65%	-	-	4.34±0.68	0.28	0.30	0.18
	Pico		2380	115	2	2%	-	-	1.71±0.19	0.22	0.22	0.07
	Nano1		2033	141	33	23%	-	-	2.6±0.4	0.28	0.28	0.11
	Nano2	M4-1	237	36	31	86%	-	-	4.52±1.13	0.29	0.28	0.10
	Pico		2603	277	7	3%	1	0%	1.61±0.2	0.18	0.18	0.06
	Nano1		1510	98	12	12%	1	1%	2.43±0.36	0.19	0.19	0.09
	Nano2	M4-2	220	34	31	91%	-	-	4.71±1.11	0.16	0.15	0.09

	Station	Number of diatoms	Nb of inactive cells	% Inactive cells	Mean diameter (μm +/- sd)	Mean division rate	Median division rate	Interquartile range (IQR)
**SIMS**	M1	81	22	27%	16.84±5.96	0.08	0.04	0.12
	M2-1	73	5	7%	12.36±7.37	0.17	0.12	0.30
	M2-2	97	26	27%	23.32±16.19	0.05	0.02	0.06
	M2-3	11	0	0%	60.89±16.28	0.12	0.09	0.14
	M4-1	36	5	14%	16.81±5.53	0.10	0.08	0.12
	M4-2	46	18	39%	14.15±5.59	0.08	0.02	0.07

Table S3: Mean amount of carbon fixed per cell, biomass and unit of carbon fixed per unit of biomass for each station and size-group. Biomass was calculated by converting the cell biovolume to carbon content according to Verity *et al.* (1992) [4].

Size-group	Station	Mean C-fixed (pgC cell^{-1})	Mean biomass (pgC cell^{-1})	Pg C-fixed / biomass
**Pico**	M2-1	0.28	1.12	0.25
	M2-2	0.23	1.09	0.21
	M2-3	0.20	0.86	0.23
	M1	0.17	1.03	0.17
	M3-1	0.08	0.54	0.15
	M4-1	0.15	1.02	0.14
	M4-2	0.11	0.88	0.12
**Nano1**	M2-1	0.55	2.55	0.22
	M2-2	0.57	2.71	0.21
	M2-3	0.62	2.58	0.24
	M1	0.39	2.85	0.14
	M3-1	0.49	3.16	0.15
	M4-1	0.54	3.10	0.17
	M4-2	0.32	2.59	0.12
**Nano2**	M2-1	0.74	7.41	0.10
	M2-2	4.37	28.10	0.16
	M2-3	3.08	17.82	0.17
	M1	1.92	18.71	0.10
	M3-1	2.01	11.63	0.17
	M4-1	2.58	14.04	0.18
	M4-2	1.39	15.27	0.09

Table S4: Compiled literature on division rates of open-ocean Sothern Ocean phytoplankton depending on iron availability. When available, standard deviation is indicated in brackets.

	Species	Division rate [d^{-1}] Fe-replete	Fe-limited	Method	Temperature	Comments	Reference
**Nano-sized flagellates**							
	Natural assemblage of small flagellates dominated by *P. antarctica*	0.37 (0.12)	0.23 (0.09)	^{13}C incubations and NanoSIMS	4-5°C	incubation of nat. communities Fe-replete corresponds to plateau samples, Fe-limited to HNLC stations	This study
Haptophytes	*P. antarctica* (strain CCMP #1871)	0.38 (0.025)	0.19 (0.006)	Chl *a* concentration	2°C	culture	[5]
	P. antarctica	0.33	0.1	cell counts		incubation of nat. communities, Antarctic Circumpolar Current	[6]
	Phaeocystis antarctica (clone AA1)	0.52	0.28	cell counts	3°C	Cultures from isolates (Polar Frontal Zone, December 2001)	[7]
	P. antarctica	0.37-0.40	0.14-0.17	cell counts	0-4°C	cultures	[8]
	Phaeocystis antarctica (clone AA1)	0.50	0.20	cell counts	3°C	cultures	[9]
	Phaeocystis antarctica (clone AA1)	0.33	0.15	fluorometry	3°C	cultures	[10]
	Phaeocystis antarctica (clone SX9)	0.41	0.14	cell counts	3°C	cultures	[9]
	P. antarctica	0.36	0.24	cell counts	2°C	cultures	[11]
	P. antarctica	0.42	0.24	cell counts	2°C	cultures	[12]
	P. antarctica	0.58	0.18	fluorometry	1°C	cultures	[13]
Cryptophytes	*Geminigera cryophila*	0.26	0.05	cell counts	2°C	cultures	[14]
**Diatoms**							
	Natural assemblage of small diatoms (<20μm)	0.24 (0.14)	0.22 (0.1)	^{13}C incubations and nanoSIMS	4-5°C	incubation of nat. communities Fe-replete corresponds to plateau samples, Fe-limited to HNLC stations	This study
	Natural assemblage of large diatoms (>20μm)	0.11 (0.14)	0.09 (0.11)	^{13}C incubations and SIMS	4-5°C	incubation of nat. communities Fe-replete corresponds to plateau samples, Fe-limited to HNLC stations	This study
	pennates	0.2	0.16	cell counts	-	incubation of nat. communities, Ross sea (oceanic)	[6]
	pennates	0.27	0.2	cell counts		incubation of nat. communities, Antarctic Circumpolar Current	[6]
	Fragilariopsis cylindrus	0.33-0.64	0.2-0.43	cell counts	0-4°C	cultures	[8]

Fragilariopsis cylindrus	0.16 (0.046)	0.05 (0.001)	Chl *a* concentration	2°C	cultures	[5]
Fragilariopsis kerguelensis	0.18	0.08	cell counts	3°C	cultures	[7]
Fragilariopsis kerguelensis	0.38	0.14	cell counts	4°C	cultures	[15]
Pseudo-nitzschia	0.67	0.27	fluorometry	1°C	cultures	[13]
Pseudo-nitzschia subcurvata	0.37-0.57	0.19-0.26	cell counts	0-4°C	cultures	[8]
Thalassiosira	0.28	0.08	cell counts	4°C	cultures	[15]
Thalassiosira antarctica	0.15	0.05	cell counts	3°C	cultures	[7]
Eucampia antarctica	0.34	0.19	cell counts	3°C	cultures	[7]
Eucampia antarctica	0.31	0.18	cell counts	3°C	cultures	[9]
Eucampia antarctica	0.26	0.14	fluorimetry	3°C	cultures	[10]
Proboscia inermis	0.47	0.29	cell counts	3°C	cultures	[7]
Proboscia inermis	0.56	0.34	cell counts	3°C	cultures	[9]
Proboscia inermis	0.44	0.30	fluorimetry	3°C	cultures	[10]
Chaetoceros	0.45-0.54	0.15-0.21	cell counts	0-4°C	cultures	[8]
Chaetoceros brevis (4-6μm)	0.39 (0.09)	0.39 (0.09)	cell counts	0-3°C	cultures	[16]
Chaetoceros dichaeta (60-80μm)	0.55	0.12	cell counts	0-3°C	cultures	[16]
Chaetoceros dichaeta	0.43	0.18	fluorometry	1°C	cultures	[13]
Chaetoceros simplex	0.5	0.23	cell counts	2°C	cultures	[14]
Chaetoceros debilis	0.37	0.19	cell counts	2°C	cultures	[12]
Actinocyclus	0.3	0.05	cell counts	4°C	cultures	[15]
Corethron pennatum	0.32	0.09	cell counts	4°C	cultures	[15]
Corethron criophilum	0.37	0.1	fluorometry	1°C	cultures	[13]
Odontella	0.23	0.03	fluorometry	1°C	cultures	[13]
**Size fractionation**						
<5μm	0.5	0.2				
5-20μm	0.23	0.05	^{14}C incubations	2.8°C	*In situ* incubations of an artificial Fe-fertilization experiment	[17]
>20μm	0.55	0.06				

1. Not F, Simon N, Biegala IC, Vaulot D. Application of fluorescent in situ hybridization coupled with tyramide signal amplification (FISH TSA) to assess eukaryotic picoplankton composition. *Aquat Microb Ecol* 2002; **28**: 157–166.
2. Simon N, Campbell L, Ornolfsdottir E, Groben R, Guillou L, Lange M, et al. Oligonucleotide probes for the identification of three algal groups by dot blot and fluorescent whole-cell hybridization. *J Eukaryot Microbiol* 2000; **47**: 76–84.
3. Not F, Latasa M, Marie D, Cariou T, Vaulot D, Simon N. A single species, *Micromonas pusilla* (Prasinophyceae), dominates the eukaryotic picoplankton in the Western English Channel. *Appl Environ Microbiol* 2004; **70**: 4064–4072.
4. Verity PG, Robertson CY, Tronzo CR, Andrews MG, Nelson JR, Sieracki ME. Relationships between cell volume and the carbon and nitrogen content of marine photosynthetic nanoplankton. *Limnol Oceanogr* 1992; **37**: 1434–1446.
5. Alderkamp A-C, Kulk G, Buma AGJ, Visser RJW, Van Dijken GL, Mills MM, et al. The effect of iron limitation on the photophysiology of *Phaeocystis antarctica* (prymnesiophyceae) and *Fragilariopsis cylindrus* (bacillariophyceae) under dynamic irradiance. *J Phycol* 2012; **48**: 45–59.
6. Coale KH, Wang X, Tanner SJ, Johnson KS. Phytoplankton growth and biological response to iron and zinc addition in the Ross Sea and Antarctic Circumpolar Current along 170°W. *Deep Sea Res Part II Top Stud Oceanogr* 2003; **50**: 635–653.
7. Strzepek RF, Maldonado MT, Hunter KA, Frew RD, Boyd PW. Adaptive strategies by Southern Ocean phytoplankton to lessen iron limitation: Uptake of organically complexed iron and reduced cellular iron requirements. *Limnol Oceanogr* 2011; **56**: 1983–2002.

8. Zhu Z, Xu K, Fu F, Spackeen JL, Bronk DA, Hutchins DA. A comparative study of iron and temperature interactive effects on diatoms and *Phaeocystis antarctica* from the Ross Sea, Antarctica. *Mar Ecol Prog Ser* 2016; **550**: 39–51.

9. Strzepek RF, Hunter KA, Frew RD, Harrison PJ, Boyd PW. Iron-light interactions differ in Southern Ocean phytoplankton. *Limnol Oceanogr* 2012; **57**: 1182–1200.

10. Strzepek RF, Boyd PW, Sunda WG. Photosynthetic adaptation to low iron, light, and temperature in Southern Ocean phytoplankton. *Proc Natl Acad Sci* 2019; **116**: 4388–4393.

11. Koch F, Beszteri S, Harms L, Trimborn S. The impacts of iron limitation and ocean acidification on the cellular stoichiometry, photophysiology, and transcriptome of *Phaeocystis antarctica*. *Limnol Oceanogr* 2019; **64**: 357–375.

12. Trimborn S, Thoms S, Bischof K, Beszteri S. Susceptibility of two Southern Ocean phytoplankton key species to iron limitation and high light. *Front Mar Sci* 2019; **6**.

13. Takeda S, Watanabe K. Growth response of Antarctic phytoplankton. 1997. pp 14–24.

14. Koch F, Trimborn S. Limitation by Fe, Zn, Co, and B12 results in similar physiological responses in two Antarctic phytoplankton species. *Front Mar Sci* 2019; **6**.

15. Timmermans KR, Wagt B van der, Baar HJW de. Growth rates, half-saturation constants, and silicate, nitrate, and phosphate depletion in relation to iron availability of four large, open-ocean diatoms from the Southern Ocean. *Limnol Oceanogr* 2004; **49**: 2141–2151.

16. Timmermans KR, Gerringa LJA, Baar HJW de, Wagt B van der, Veldhuis MJW, Jong JTM de, et al. Growth rates of large and small Southern Ocean diatoms in relation to availability o iron in natural seawater. *Limnol Oceanogr* 2001; **46**: 260–266.

17. Gall MP, Strzepek R, Maldonado M, Boyd PW. Phytoplankton processes. Part 2: Rates of primary production and factors controlling algal growth during the Southern Ocean Iron RElease Experiment (SOIREE). *Deep Sea Res Part II Top Stud Oceanogr* 2001; **48**: 2571–2590.
